# Supplementary material for: Genome-wide CRISPR Screens in T Helper Cells Reveal Pervasive Crosstalk between Activation and Differentiation
Source: Cell. 2019 Feb 7;176(4):882–896.e18. doi: 10.1016/j.cell.2018.11.044 (PMC6370901; doi:10.1016/j.cell.2018.11.044)
Supplement: Data S2. Processed Data from All the Steps of the Analysis, Related to Figure 1 [file mmc2.zip › supplemental data/motif analysis/Bhlhe40_homer/homerResults/motif11.similar.html]

motif11

## Information for motif11

A
C
G
T
A
G
T
C
C
A
G
T
A
G
T
C
A
C
T
G
G
T
A
C
A
C
G
T
C
G
T
A
  
Reverse Opposite:  

A
C
G
T
G
T
C
A
A
C
T
G
A
G
T
C
A
C
T
G
G
T
C
A
T
C
A
G
C
G
T
A
  

|  |  |
| --- | --- |
| p-value: | 1e-10 |
| log p-value: | -2.458e+01 |
| Information Content per bp: | 1.839 |
| Number of Target Sequences with motif | 50.0 |
| Percentage of Target Sequences with motif | 4.17% |
| Number of Background Sequences with motif | 590.3 |
| Percentage of Background Sequences with motif | 1.39% |
| Average Position of motif in Targets | 118.3 +/- 53.6bp |
| Average Position of motif in Background | 94.2 +/- 68.7bp |
| Strand Bias (log2 ratio + to - strand density) | 0.0 |
| Multiplicity (# of sites on avg that occur together) | 1.06 |
| Motif File: | file (matrix) reverse opposite |

### Similar de novo motifs found

|  |  |  |  |  |  |  |  |
| --- | --- | --- | --- | --- | --- | --- | --- |
| Rank | Match Score | Redundant Motif | P-value | log P-value | % of Targets | % of Background | Motif file |
| 1 | 0.707 | G A T C A C G T A G T C A T C G G A T C A C T G C G T A A C T G C G T A C T A G | 1e-6 | -14.733250 | 1.92% | 0.55% | motif file (matrix) |
| 2 | 0.682 | A G C T T A G C A G C T A T G C A C T G A T G C A C T G G C T A C A T G C G T A T A C G A C G T | 1e-6 | -14.205515 | 1.50% | 0.36% | motif file (matrix) |
